# Association between maternal glucose levels in pregnancy and offspring’s metabolism and adiposity: an 18-year birth cohort study

**DOI:** 10.1007/s00125-025-06476-6

**Published:** 2025-07-02

**Authors:** Yuzhi Deng, Hanbin Wu, Noel Y. H. Ng, Claudia H. T. Tam, Atta Y. T. Tsang, Michael H. M. Chan, Kenneth Ka Hei Lo, Chi Chiu Wang, Wing Hung Tam, Ronald C. W. Ma

**Affiliations:** 1 Department of Medicine and Therapeutics, The Chinese University of Hong Kong, Shatin, Hong Kong, China; 2 Department of Obstetrics and Gynaecology, The Chinese University of Hong Kong, Shatin, Hong Kong, China; 3 Department of Chemical Pathology, The Chinese University of Hong Kong, Shatin, Hong Kong, China; 4 Department of Food Science and Nutrition, The Hong Kong Polytechnic University, Hung Hom, Hong Kong, China; 5 Li Ka Shing Institute of Health Sciences, The Chinese University of Hong Kong, Shatin, Hong Kong, China; 6 CUHK Medical Centre, Shatin, Hong Kong, China; 7 Hong Kong Institute of Diabetes and Obesity, The Chinese University of Hong Kong, Shatin, Hong Kong, China

**Keywords:** Birth cohort, Gestational diabetes mellitus, Hyperglycaemia, Long-term association

## Abstract

**Aims/hypothesis:**

The study aimed to explore the association between maternal glucose levels in pregnancy and offspring’s metabolism and adiposity at approximately 18 years of age.

**Methods:**

Pregnant women from the Hong Kong Field Centre enrolled in the Hyperglycemia and Adverse Pregnancy Outcome (HAPO) study underwent a 75 g OGTT at 24–32 gestational weeks. Offspring’s metabolic and adiposity traits were assessed at 18 years postpartum. Associations were evaluated using multiple linear regression and logistic regression.

**Results:**

Among the 506 mother–child pairs followed up to 18 years, maternal fasting plasma glucose (FPG) in pregnancy was positively associated with offspring’s FPG (β = 0.06 [95% CI 0.02, 0.09]), while maternal 1 h plasma glucose (PG) showed a positive association with offspring’s FPG (β = 0.05), 30 min PG (β = 0.21) and 2 h PG (β = 0.14). All maternal glycaemic levels were associated with an increased risk of offspring being overweight/obese, particularly maternal 1 h PG (OR 1.50 [95% CI 1.17, 1.93]). Offspring of mothers with gestational diabetes mellitus showed a higher prevalence of abnormal glucose tolerance (11.86% vs 7.97%), impaired fasting glucose (1.89% vs 0.49%) and impaired glucose tolerance (10.34% vs 7.13%) than offspring of mothers with normal glucose tolerance, although these associations did not reach statistical significance in fully adjusted models, underscoring the benefit of considering maternal glucose as a continuous trait.

**Conclusions/interpretation:**

Maternal glucose levels in pregnancy showed a long-term association with offspring’s metabolic health into young adulthood, with continuous associations across the full maternal glucose spectrum, suggesting a graded effect of maternal hyperglycaemia on offspring’s metabolic risk.

**Graphical Abstract:**

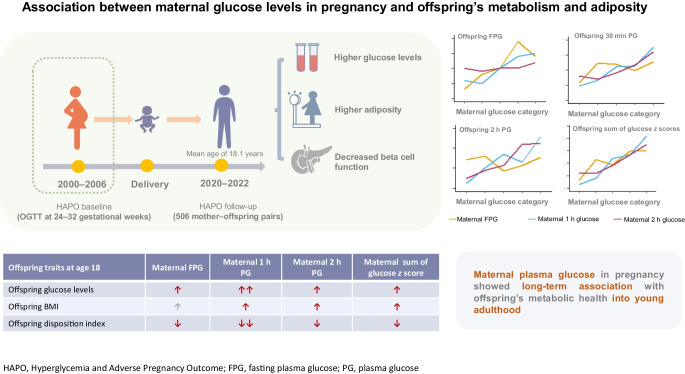

**Supplementary Information:**

The online version contains peer-reviewed but unedited supplementary material available at 10.1007/s00125-025-06476-6.


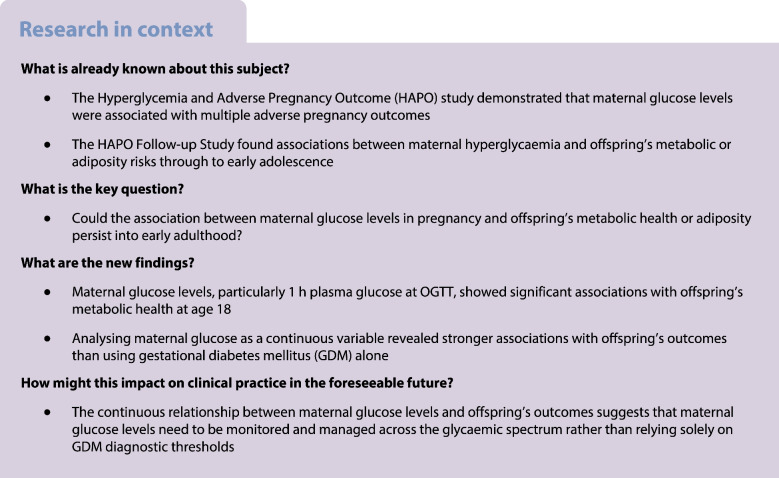


## Introduction

The global prevalence of gestational diabetes mellitus (GDM) has risen substantially in recent decades, paralleling worldwide increases in obesity and type 2 diabetes [1]. According to the International Diabetes Federation’s Diabetes Atlas, in 2021 GDM affected approximately 14.0% of pregnancies globally [2]. Women with GDM experience increased risks of pregnancy complications and subsequent type 2 diabetes mellitus, while their offspring face both immediate and long-term health consequences, including macrosomia, clinical neonatal hypoglycaemia, childhood obesity and an increased risk of metabolic disorders [3]. These associations are particularly concerning, as growing evidence suggests that intrauterine exposure to maternal hyperglycaemia may have long-lasting effects on offspring’s health throughout their life course.

These concerning trends have prompted extensive research into the relationship between maternal glucose levels and offspring’s outcomes. The Hyperglycemia and Adverse Pregnancy Outcome (HAPO) study, an observational multinational cohort study, demonstrated that maternal glycaemic levels in pregnancy, below those diagnostic of diabetes, were associated with adverse pregnancy outcomes [4]. Based on these findings, the International Association of the Diabetes and Pregnancy Study Groups (IADPSG) proposed new diagnostic criteria for GDM, which were subsequently adopted by WHO [5]. After completion of the original HAPO study, the Hong Kong Field Centre conducted a follow-up study at 7 years, showing that maternal hyperglycaemia during pregnancy increased offspring’s risk of abnormal glucose tolerance (AGT) and obesity in early childhood [6]. Subsequently, the multinational HAPO Follow-up Study (HAPO-FUS) evaluated 4697 women and 4832 offspring at a median age of 11.4 years postpartum, revealing higher obesity prevalence among offspring of mothers with GDM and an independent association between maternal plasma glucose and offspring’s glucose metabolism [7].

While these studies have established clear associations between maternal plasma glucose and offspring’s health through early adolescence, whether these correlations persist into late adolescence and early adulthood remains poorly understood [8]. The transition to late adolescence or early adulthood represents a crucial developmental window for metabolic health, as it marks a time when metabolic disorders often emerge and lifestyle patterns that influence long-term health are established [9]. Understanding the persistent effects of maternal plasma glucose beyond early adolescence is therefore crucial for developing effective prevention strategies. This study aims to investigate the long-term association between maternal glucose levels during pregnancy and offspring’s metabolism and adiposity in early adulthood, examining these associations across the full range of maternal glucose values to inform targeted prevention strategies.

## Methods

### Study design

This prospective cohort study followed mother–child pairs from the Hong Kong Field Centre of the Hyperglycemia and Adverse Pregnancy Outcome (HAPO) study. The original HAPO study was a multicentre observational population-based study investigating associations between maternal glucose levels and perinatal outcomes [4]. The source population consisted of pregnant women at the Prince of Wales Hospital in Hong Kong. Eligibility criteria for the HAPO population have been described previously [4]. Initially, 1667 pregnant women with singleton pregnancies were enrolled in the Hong Kong Field Centre (2000–2006), and they all underwent a 75 g OGTT at 24–32 gestational weeks. Demographic characteristics, including maternal education level, parity, smoking/alcohol habits and family history, were collected using standardised questionnaires. The OGTT results were blinded for both participants and clinicians unless fasting plasma glucose (FPG) was >5.8 mmol/l and/or 2 h plasma glucose (PG) was >11.1 mmol/l, or either glucose measurement was <2.5 mmol/l. Of the initial 1667 participants in the Hong Kong HAPO study, exclusion criteria included non-Asian participants and those whose OGTT results were unblinded. Eligible participants were invited to a follow-up assessment at the Prince of Wales Hospital between 2020 and 2022, at approximately 18 years postpartum. Finally, a total of 506 mother–child pairs were followed up and included in our analysis. To assess potential selection bias, we compared baseline characteristics between participants in the follow-up and those lost to follow-up (electronic supplementary material [ESM] Table 1). The original HAPO Hong Kong cohort consisted primarily of Chinese women aged 18-45 years with varied educational backgrounds, generally representing the pregnant population in Hong Kong.

The study was approved by the joint New Territories East Cluster (NTEC) and Chinese University of Hong Kong (CUHK) clinical research ethics committee. The study was reported according to the Strengthening the Reporting of Observational Studies in Epidemiology (STROBE) checklist for cohort studies (ESM Fig. 1).

### Data collection

Both the mother and her child attended a follow-up visit in the morning after at least 8 h of fasting. We collected updated demographic and lifestyle characteristics of the offspring, including educational level, smoking/alcohol habits, family history, medical history, physical activities patterns and dietary habits. Physical activity was recorded by intensity and frequency, while dietary intake was assessed using a food frequency questionnaire.

Anthropometric parameters and BP measurements were taken using standardised procedures. Height was measured to the nearest 0.1 cm with a Harpenden stadiometer (Holtain, Crymych, UK), and weight was recorded to the nearest 0.1 kg using a Tanita digital physician scale (Tanita, Tokyo, Japan). Waist circumference was measured at the narrowest point between the xiphisternum and umbilicus, while the hip circumference was taken as the horizontal measure around the pelvis at the point of maximal protrusion of the buttocks. BP was measured three times in the non-dominant arm using an Omron device (Omron Healthcare, Kyoto, Japan) and the mean readings were used for analysis. Body fat percentage was measured using a bioelectrical impedance analysis system (Tanita, Tokyo, Japan). All participants underwent a 75 g OGTT. Blood samples were collected from offspring at fasting, 30 min and 2 h time points to determine PG and insulin levels. Samples were preserved at −80℃ for subsequent analysis.

### Laboratory measurements

Laboratory assessments employed a hexokinase method for PG (Hitachi 911; Boehringer Mannheim, Mannheim, Germany) and automated ion-exchange HPLC for HbA_1c_. Insulin was determined by electro-chemiluminescent immunoassay on a Roche Cobas E411 analyser (Roche Diagnostics, Indianapolis, IN, USA), and enzymatic techniques were used for lipid profiling.

### Exposure, outcome and covariates

The primary exposures were maternal FPG, 1 h PG and 2 h PG during the HAPO pregnancy OGTT and an integrated measure using the sum of their *z* scores. The sum of individual glucose *z* scores was calculated for each OGTT time point by subtracting the mean glucose level from all observed values at each time point, dividing by the SD of the glucose values at that time point and summing the four individual *z* scores. Secondary exposure was GDM, which was defined post hoc using the IADPSG criteria: fasting blood glucose values ≥5.1 mmol/l, 1 h glucose values ≥10.0 mmol/l or 2 h glucose values ≥8.5 mmol/l [5].

Primary offspring outcomes focused on glucose intolerance and adiposity. Glucose intolerance was defined as follows: impaired fasting glucose (IFG [FPG 5.6–6.9 mmol/l]), impaired glucose tolerance (IGT [2 h glucose 7.8–11.0 mmol/l]) and abnormal glucose tolerance (AGT [FPG ≥5.6 mmol/l and/or 2 h PG ≥7.8 mmol/l]). Adiposity was mainly assessed by determining if the participant was overweight/obese using BMI, with categorisation defined by the National Health and Family Planning Commission of China for the Chinese population (≥24 kg/m^2^) [10]. Additional adiposity indicators included waist circumference (≥90 cm for men, ≥80 cm for women) [11], hip circumference, waist/hip ratio (≥0.90 for men, ≥0.85 for women) [12] and body fat percentage (≥25% for men, ≥35% for women) based on WHO guidelines [13]. All of these adiposity measures were also evaluated using the 85th percentile of our study population as alternative cut-off points.

Additional outcomes included continuous glucose levels from offspring’s OGTT and measures of insulin sensitivity and beta cell function. These were estimated using the Matsuda index, insulinogenic index and oral disposition index. The Matsuda index was calculated using the following formula: [10,000/square root of (PG_0 min_ × insulin_0 min_) × (PG_mean_ × insulin_mean_)]. The insulinogenic index was defined as the ratio of incremental insulin to the incremental glucose during the first 30 min of the OGTT: [Insulin_30 min_ – insulin_0 min_]/[PG_30 min_ – PG_0 min_]. The oral disposition index, which assesses the acute insulin response in relation to the level of insulin sensitivity, was calculated as the product of the Matsuda and insulinogenic indices and log transformed. Binary outcomes included glucose levels above the 85th percentile and insulin sensitivity indices below the 15th percentile to identify individuals with significant metabolic alterations.

Maternal and offspring’s characteristics that could potentially affect outcomes were considered as potential confounders, including maternal age at OGTT, pre-pregnancy BMI, education level (categorised as ‘higher’ for over 11 years of education or ‘lower’ for 10 years or fewer), parity (primiparity/multipara), maternal smoking (yes/no), offspring’s sex (male/female), offspring’s BMI and offspring’s physical activity (moderate to vigorous intensity physical activity (MVPA) ≥150 min/week or not). Offspring’s dietary intake was assessed using a food frequency questionnaire validated in the Hong Kong community [14], then the total Diet Quality Index-International (DQI-I) was computed to reflect the overall dietary quality.

### Data analysis

Descriptive statistics were calculated, with categorical variables summarised as frequencies and counts, and continuous variables as means and SD. Baseline characteristics were compared between the group with normal glucose tolerance (NGT) and the group with GDM. Maternal PG levels (fasting, 1 h and 2 h) at OGTT in pregnancy were divided into quintiles. The measurements of offspring’s metabolic parameters at age 18 were calculated as means for each quintile. Trend lines were constructed using these means to visualise potential linear relationships between maternal glucose levels in pregnancy and offspring’s glucose metabolism and beta cell function. Multiple linear regression was used for continuous outcomes, while multivariable logistic regression was applied for binary outcomes to evaluate associations with maternal glucose levels. Three progressive adjustment models were constructed: model 1 (basic model) adjusted only for offspring’s age and sex, model 2 (confounder model) additionally adjusted for maternal confounder factors (age at OGTT, pre-pregnancy BMI, education, parity and smoking) and model 3 (precision model) additionally adjusted for offspring’s BMI, physical activity and DQI-I based on model 2. Missing data in covariates were not imputed in our analyses, due to them accounting for a very small proportion (less than 5%), which was not likely to have a significant impact on our results. The primary analysis was conducted using a significance threshold of *p*<0.05 (two sided).

To address the multiple comparison issue while maintaining adequate statistical power, we additionally employed the Benjamini–Hochberg procedure to control the false discovery rate (FDR) at 5%. The FDR adjustments were applied separately for each exposure group (maternal fasting glucose, 1 h glucose, 2 h glucose and the sum of all glucose *z* scores) among all adjusted models to maintain the biological independence of different glucose measurements. All data analyses were performed using R software (version 4.3.2; www.r-project.org/).

## Results

### Participants

Of the 1612 eligible mother–child pairs, 506 participated in the follow-up assessment. Baseline characteristics comparing those who attended the follow-up and those lost to follow-up are shown in ESM Table 1. Among participants, 59 mothers (11.7%) had GDM, with diagnostic criteria met through elevated FPG (*n*=10, 17.0%), 1 h PG (*n*=38, 64.4%) and 2 h PG (*n*=42, 71.2%), respectively.

Baseline and follow-up characteristics (ESM Table 2) revealed that mothers with GDM were significantly older and had higher pre-conceptional BMI than mothers with NGT (*p*<0.05). At the 18-year follow-up, mothers with GDM maintained higher FPG levels, HbA_1c_ levels and fasting insulin levels and had a fivefold higher risk of type 2 diabetes than mothers without GDM (OR 5.17 [95% CI 2.10, 12.32]). After adjusting for maternal age, pre-pregnancy BMI, education level (higher/lower), parity (primiparity/multipara), smoking status at OGTT (yes/no), and current BMI (model 2), the OR was increased to 5.39 (95% CI 1.97, 14.17) (ESM Table 3).

The mean age of offspring at follow-up was 18.09 ± 0.77 years (47.8% men). Their metabolic parameters, including fasting glucose, insulin and lipid profiles, were similar between offspring of mothers with GDM and those with NGT.

### Maternal glucose levels and offspring’s glucose metabolism

**Fig. 1:**
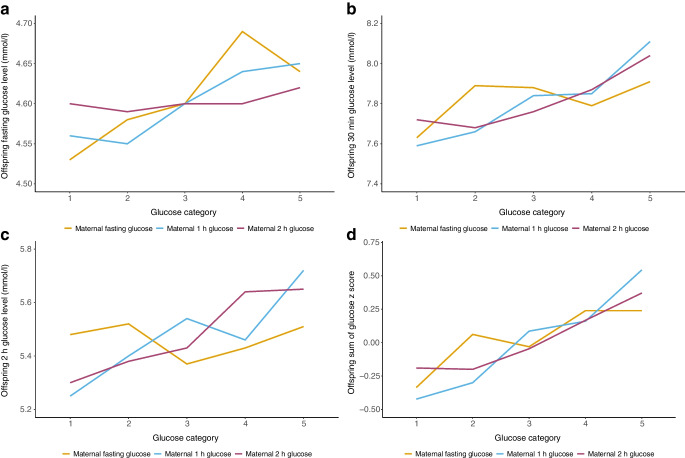
Offspring glucose levels at 18 years old across five categories of maternal glucose levels. Mean levels of offspring’s (**a**) fasting glucose level, (**b**) 30 min glucose level, (**c**) 2 h glucose level and (**d**) sum of glucose *z* scores at OGTT across categories of maternal fasting, 1 h and 2 h glucose levels at OGTT in pregnancy

The analysis of maternal glucose quintile categories shows a positive association with offspring’s mean FPG, 30 min PG and 2 h PG, as well as with the sum of all glucose *z* scores (Fig. 1). This relationship persisted after adjusting for potential covariates in a multiple linear regression model (Table 1). Each SD increase in maternal fasting glucose level was consistently associated with higher offspring’s fasting glucose level (β = 0.06 [95% CI 0.02, 0.09]), with the strongest association observed between maternal 1 h glucose level and offspring’s 30 min glucose level (β = 0.21 [95% CI 0.09, 0.33]) and between the former and glucose *z* scores (β = 0.39 [95% CI 0.19, 0.60]). Maternal 2 h glucose levels showed weaker associations with the sum of offspring’s glucose *z* scores (β = 0.24 [95% CI 0.03, 0.46]).

**Table 1:** Association between continuous maternal glucose level and offspring’s glucose traits at 18 years old

Variable	Model 1		Model 2		Model 3	
	β (95% CI)	*p* value	β (95% CI)	*p* value	β (95% CI)	*p* value
Maternal fasting glucose at OGTT						
Fasting glucose (mmol/l)	0.05 (0.02, 0.08)	0.001*	0.06 (0.03, 0.09)	<0.001*	0.06 (0.02, 0.09)	0.001*
30 min glucose (mmol/l)	0.11 (0.00, 0.22)	0.061	0.12 (0.00, 0.23)	0.051	0.09 (−0.03, 0.21)	0.145
2 h glucose (mmol/l)	0.003 (−0.123, 0.129)	0.965	0.007 (−0.125, 0.139)	0.918	−0.053 (−0.181, 0.076)	0.422
Sum of glucose *z* scores	0.24 (0.04, 0.43)	0.017*	0.26 (0.06, 0.47)	0.012*	0.21 (−0.01, 0.41)	0.047
Maternal 1 h glucose level at OGTT						
Fasting glucose (mmol/l)	0.04 (0.01, 0.07)	0.006*	0.05 (0.02, 0.08)	0.002*	0.05 (0.01, 0.08)	0.007*
30 min glucose (mmol/l)	0.20 (0.09, 0.31)	0.001*	0.21 (0.09, 0.32)	0.001*	0.21 (0.09, 0.33)	0.001*
2 h glucose (mmol/l)	0.13 (0.01, 0.26)	0.035*	0.18 (0.05, 0.31)	0.007*	0.14 (0.01, 0.27)	0.034*
Sum of glucose *z* scores	0.36 (0.17, 0.56)	<0.001*	0.43 (0.22, 0.63)	<0.001*	0.39 (0.19, 0.60)	<0.001*
Maternal 2 h glucose level at OGTT						
Fasting glucose (mmol/l)	0.01 (−0.02, 0.04)	0.373	0.02 (−0.01, 0.05)	0.194	0.02 (−0.02, 0.05)	0.365
30 min glucose (mmol/l)	0.14 (0.03, 0.25)	0.016*	0.14 (0.02, 0.26)	0.027*	0.14 (0.01, 0.26)	0.033*
2 h glucose (mmol/l)	0.12 (−0.01, 0.24)	0.065	0.15 (0.02, 0.29)	0.026*	0.13 (−0.01, 0.26)	0.064
Sum of glucose *z* scores	0.22 (0.03, 0.42)	0.024*	0.27 (0.06, 0.48)	0.011*	0.24 (0.03, 0.46)	0.029*
Maternal sum of all glucose *z* scores						
Fasting glucose (mmol/l)	0.02 (0.01, 0.03)	0.003*	0.02 (0.01, 0.04)	0.001*	0.02 (0.01, 0.04)	0.002*
30 min glucose (mmol/l)	0.08 (0.03, 0.13)	0.001*	0.09 (0.04, 0.14)	0.001*	0.08 (0.03, 0.13)	0.003*
2 h glucose (mmol/l)	0.05 (−0.01, 0.10)	0.091	0.06 (0.01, 0.12)	0.028*	0.04 (−0.02, 0.10)	0.185
Sum of glucose *z* scores	0.15 (0.07, 0.23)	<0.001*	0.18 (0.09, 0.27)	<0.001*	0.15 (0.06, 0.24)	0.001*

β values represent the change in offspring’s glucose traits for each SD increase of maternal glucose measures at OGTT: fasting glucose 0.31 mmol/l, 1 h glucose 1.58 mmol/l and 2 h glucose 1.27 mmol/l

Model 1: offspring’s age and offspring’s sex (male/female)

Model 2: model 1 + maternal age, maternal pre-pregnancy BMI, maternal education level (higher/lower), parity (primiparity/multipara) and maternal smoking (yes/no)

Model 3: model 2 + offspring’s BMI, offspring’s physical activity (MVPA ≥150 min/week or not) and total DQI-I

^*^p<0.05 (after adjusted using the Benjamini–Hochberg procedure with FDR = 0.05)

Further categorical analysis (ESM Table 4) revealed that, when using established diagnostic thresholds for glucose impairment, maternal 1 h glucose levels were strongly associated with a higher risk of offspring’s IFG (OR 3.37 [95% CI 1.12, 11.66]). Using population-based 85th percentile cut-offs, we found consistent associations between maternal glucose levels and offspring’s glucose traits, particularly for the sum of offspring’s glucose *z* scores across all maternal OGTT time points (fasting OR 1.35 [95% CI 1.03, 1.74], 1 h OR 1.84 [95% CI 1.39, 2.47], 2 h OR 1.43 [95% CI 1.08, 1.90]).

The associations between maternal GDM status and offspring’s glucose traits were less significant than those observed with continuous measures of maternal glucose. While offspring born to mothers with GDM demonstrated a higher prevalence of AGT (11.86% vs 7.97%), IFG (1.89% vs 0.49%) and IGT (10.34% vs 7.13%) than offspring of mothers with NGT, these differences did not reach statistical significance in fully adjusted models (AGT OR 1.45 [95% CI 0.50, 3.63], IFG OR 6.66 [95% CI 0.25, 124.18], IGT OR 1.35 [95% CI 0.42, 3.62]) (ESM Table 5).

### Maternal glucose levels and offspring’s adiposity traits

The prevalence of overweight/obesity was higher among offspring of mothers with GDM (23.73%, 14/59) than those born to mothers with NGT (20.67%, 92/445). Maternal plasma glucose showed significant associations with offspring’s adiposity traits (Table 2). In fully adjusted models, each SD mmol/l increase in maternal fasting glucose level was associated with increased odds of offspring being overweight/obese (OR 1.32 [95% CI 1.04, 1.69]). Maternal 1 h glucose showed the most consistent and robust associations, with each SD mmol/l increase positively associated with offspring being overweight/obese (OR 1.50 [95% CI 1.17, 1.93]). While these associations were slightly attenuated in model 3, the maternal sum of glucose *z* scores maintained a significant prediction of offspring’s BMI at approximately 18 years of age (ESM Table 6), suggesting a cumulative relationship between maternal glucose levels and offspring’s adiposity measures. Although the offspring of mothers with GDM showed a trend towards an increased risk of elevated adiposity traits, most of these associations did not reach statistical significance in the fully adjusted models (ESM Table 7).

**Table 2:** Association between maternal glucose level at OGTT and offspring’s adiposity traits at 18 years old

Variable	*n* (%) *N*=504	Model 1		Model 2		Model 3	
		OR (95% CI)	*p* value	OR (95% CI)	*p* value	OR (95% CI)	*p* value
Maternal fasting glucose level at OGTT							
Overweight/obesity^†^	106 (21.03)	1.33 (1.07, 1.66)	0.009*	1.28 (1.01, 1.62)	0.042*	1.32 (1.04, 1.69)	0.023*
Waist circumference above WHO cut-off values	67 (13.29)	1.38 (1.07, 1.77)	0.011*	1.34 (1.02, 1.75)	0.033*	1.17 (0.74, 1.91)	0.498
Waist circumference above 85th percentile	73 (14.48)	1.32 (1.02, 1.71)	0.031*	1.37 (1.03, 1.81)	0.029*	2.23 (1.13, 4.95)	0.031
Hip circumference above 85th percentile	76 (15.08)	1.33 (1.04, 1.69)	0.022*	1.35 (1.04, 1.75)	0.023*	1.46 (0.90, 2.44)	0.136
Waist/hip ratio above WHO cut-off values	71 (14.09)	1.32 (1.03, 1.69)	0.024*	1.31 (1.01, 1.69)	0.043	1.27 (0.95, 1.71)	0.105
Waist/hip ratio above 85th percentile	76 (15.08)	1.31 (1.02, 1.69)	0.031*	1.33 (1.02, 1.74)	0.036	1.30 (0.96, 1.78)	0.090
Body fat percentage above WHO cut-off values	118 (23.41)	1.20 (0.97, 1.48)	0.095	1.12 (0.90, 1.41)	0.310	0.95 (0.67, 1.35)	0.770
Body fat percentage above 85th percentile	76 (15.08)	1.32 (1.04, 1.68)	0.021*	1.26 (0.98, 1.63)	0.074	1.16 (0.80, 1.68)	0.421
Maternal 1 h glucose level at OGTT							
Overweight/obesity^†^	106 (21.03)	1.47 (1.17, 1.84)	0.001*	1.52 (1.20, 1.93)	0.001*	1.50 (1.17, 1.93)	0.002*
Waist circumference above WHO cut-off values	67 (13.29)	1.27 (0.98, 1.64)	0.072	1.30 (0.99, 1.72)	0.058	1.03 (0.59, 1.75)	0.923
Waist circumference above 85th percentile	73 (14.48)	1.32 (1.01, 1.72)	0.039	1.46 (1.11, 1.95)	0.008*	1.35 (0.73, 2.67)	0.354
Hip circumference above 85th percentile	76 (15.08)	1.41 (1.10, 1.82)	0.007*	1.49 (1.14, 1.95)	0.003*	1.29 (0.78, 2.19)	0.330
Waist/hip ratio above WHO cut-off values	71 (14.09)	1.05 (0.82, 1.35)	0.687	1.07 (0.82, 1.40)	0.616	0.92 (0.65, 1.27)	0.601
Waist/hip ratio above 85th percentile	76 (15.08)	1.09 (0.85, 1.41)	0.485	1.14 (0.87, 1.49)	0.333	1.00 (0.73, 1.38)	0.985
Body fat percentage above WHO cut-off values	118 (23.41)	1.17 (0.94, 1.45)	0.148	1.17 (0.93, 1.47)	0.182	0.85 (0.60, 1.21)	0.372
Body fat percentage above 85th percentile	76 (15.08)	1.23 (0.97, 1.57)	0.089	1.25 (0.96, 1.63)	0.098	1.04 (0.68, 1.57)	0.848
Maternal 2 h glucose level at OGTT							
Overweight/obesity^†^	106 (21.03)	1.44 (1.15, 1.80)	0.001*	1.53 (1.20, 1.96)	0.001*	1.50 (1.17, 1.95)	0.002*
Waist circumference above WHO cut-off values	67 (13.29)	1.22 (0.94, 1.58)	0.126	1.32 (0.99, 1.75)	0.058	0.71 (0.38, 1.24)	0.240
Waist circumference above 85th percentile	73 (14.48)	1.36 (1.04, 1.78)	0.025	1.60 (1.19, 2.16)	0.002*	1.27 (0.65, 2.56)	0.490
Hip circumference above 85th percentile	76 (15.08)	1.58 (1.23, 2.04)	<0.001*	1.75 (1.33, 2.32)	<0.001*	1.94 (1.12, 3.42)	0.019
Waist/hip ratio above WHO cut-off values	71 (14.09)	1.07 (0.84, 1.37)	0.565	1.14 (0.87, 1.50)	0.338	0.96 (0.68, 1.33)	0.795
Waist/hip ratio above 85th percentile	76 (15.08)	1.15 (0.89, 1.49)	0.288	1.25 (0.95, 1.66)	0.114	1.05 (0.75, 1.47)	0.787
Body fat percentage above WHO cut-off values	118 (23.41)	1.16 (0.94, 1.44)	0.173	1.18 (0.93, 1.49)	0.177	0.77 (0.52, 1.12)	0.174
Body fat percentage above 85th percentile	76 (15.08)	1.13 (0.89, 1.44)	0.304	1.19 (0.91, 1.56)	0.196	0.87 (0.58, 1.30)	0.502
Maternal sum of all glucose *z* scores							
Overweight/obesity^†^	106 (21.03)	1.20 (1.09, 1.32)	<0.001*	1.22 (1.10, 1.36)	<0.001*	1.22 (1.10, 1.36)	<0.001*
Waist circumference above WHO cut-off values	67 (13.29)	1.14 (1.03, 1.27)	0.010*	1.17 (1.04, 1.31)	0.012*	0.99 (0.79, 1.24)	0.913
Waist circumference above 85th percentile	73 (14.48)	1.17 (1.04, 1.30)	0.001*	1.24 (1.09, 1.40)	0.001*	1.26 (0.97, 1.70)	0.099
Hip circumference above 85th percentile	76 (15.08)	1.21 (1.09, 1.35)	<0.001*	1.26 (1.12, 1.41)	<0.001*	1.27 (1.02, 1.62)	0.041
Waist/hip ratio above WHO cut-off values	71 (14.09)	1.07 (0.97, 1.19)	0.128	1.09 (0.97, 1.23)	0.128	1.03 (0.90, 1.18)	0.668
Waist/hip ratio above 85th percentile	76 (15.08)	1.10 (0.98, 1.22)	0.045	1.13 (1.00, 1.26)	0.045	1.06 (0.93, 1.21)	0.380
Body fat percentage above WHO cut-off values	118 (23.41)	1.09 (1.00, 1.19)	0.111	1.08 (0.98, 1.20)	0.111	0.92 (0.78, 1.07)	0.277
Body fat percentage above 85th percentile	76 (15.08)	1.11 (1.01, 1.23)	0.040	1.13 (1.00, 1.26)	0.040	1.01 (0.85, 1.20)	0.868

ORs represent the risk of offspring adiposity traits per SD mmol/l increase in maternal glucose level at OGTT: fasting glucose 0.31 mmol/l, 1 h glucose 1.58 mmol/l and 2 h glucose 1.27 mmol/l. Offspring obesity-related outcomes were defined according to sex-specific cut-off points: adiposity^†^ (overweight/obesity using WHO criteria), waist circumference (≥90 cm for men, ≥80 cm for women), waist/hip ratio (≥0.9 for men, ≥0.8 for women) and body fat percentage (≥25% for men, ≥30% for women). The 85th percentile cut-offs were determined from the study population distribution

Model 1: offspring’s age and offspring’s sex (male/female)

Model 2: model 1 + maternal age, maternal pre-pregnancy BMI, maternal education level (higher/lower), parity (primiparity/multipara) and maternal smoking (yes/no)

Model 3: model 2 + offspring’s BMI and offspring’s physical activity (MVPA ≥150 min/week or not) and total DQI-I

^†^Overweight/obesity was defined as BMI ≥24 kg/m^2^. Analysis was not adjusted for offspring’s BMI

^*^p<0.05 (after adjusted using the Benjamini–Hochberg procedure with FDR = 0.05)

### Maternal glucose levels and offspring’s insulin resistance/beta cell function

Offspring’s insulinogenic and oral disposition indices, as measures of beta cell function, showed declining trends across increasing categories of maternal glucose level during pregnancy OGTT (ESM Fig. 2). Among the maternal glucose measurements, 1 h glucose showed the strongest associations with offspring’s insulin resistance/beta cell function parameters, with each SD mmol/l increase significantly associated with higher odds of a low insulinogenic index (OR 1.59 [95% CI 1.19, 2.13]) and disposition index (OR 1.48 [95% CI 1.13, 1.96]) (Table 3, ESM Table 8). These findings suggest a potential long-term association between maternal plasma glucose and offspring’s beta cell function that may involve different mechanistic pathways.

**Table 3:** Association between maternal glucose level during OGTT and offspring’s insulin sensitivity/beta cell function traits at 18 years old

Variable	*n*/*N* (%)	Model 1		Model 2		Model 3	
		OR (95% CI)	*p* value	OR (95% CI)	*p* value	OR (95% CI)	*p* value
Maternal fasting glucose level at OGTT							
Insulinogenic index below 15th percentile	74/491 (15.07)	0.95 (0.74, 1.22)	0.713	0.94 (0.72, 1.22)	0.672	0.89 (0.66, 1.20)	0.455
Matsuda index below 15th percentile	75/495 (15.15)	1.10 (0.86, 1.40)	0.437	1.08 (0.83, 1.40)	0.542	0.96 (0.71, 1.28)	0.769
Disposition index below 15th percentile	74/491 (15.07)	1.15 (0.9, 1.47)	0.253	1.17 (0.91, 1.50)	0.226	1.14 (0.88, 1.47)	0.313
Maternal 1 h glucose level at OGTT							
Insulinogenic index below 15th percentile	74/491 (15.07)	1.31 (1.02, 1.69)	0.032*	1.34 (1.03, 1.74)	0.028*	1.59 (1.19, 2.13)	0.002*
Matsuda index below 15th percentile	75/495 (15.15)	1.18 (0.93, 1.51)	0.176	1.26 (0.97, 1.64)	0.084	1.10 (0.80, 1.50)	0.560
Disposition index below 15th percentile	74/491 (15.07)	1.46 (1.14, 1.87)	0.003*	1.50 (1.16, 1.95)	0.002*	1.48 (1.13, 1.96)	0.005*
Maternal 2 h glucose level at OGTT							
Insulinogenic index below 15th percentile	74/491 (15.07)	1.24 (0.96, 1.60)	0.095	1.24 (0.94, 1.62)	0.122	1.41 (1.05, 1.90)	0.023
Matsuda index below 15th percentile	75/495 (15.15)	1.12 (0.88, 1.43)	0.369	1.24 (0.94, 1.63)	0.115	1.03 (0.75, 1.41)	0.847
Disposition index below 15th percentile	74/491 (15.07)	1.29 (1.01, 1.66)	0.040	1.36 (1.04, 1.77)	0.024	1.30 (0.98, 1.71)	0.065
Maternal sum of all glucose *z* scores							
Insulinogenic index below 15th percentile	74/491 (15.07)	1.08 (0.97, 1.20)	0.149	1.08 (0.97, 1.21)	0.159	1.13 (1.00, 1.28)	0.045
Matsuda index below 15th percentile	75/495 (15.15)	1.07 (0.96, 1.18)	0.204	1.10 (0.98, 1.23)	0.092	1.01 (0.89, 1.15)	0.846
Disposition index below 15th percentile	74/491 (15.07)	1.14 (1.03, 1.27)	0.010*	1.17 (1.05, 1.30)	0.006*	1.15 (1.02, 1.29)	0.017*

ORs represent the risk of lower offspring beta cell function traits per SD mmol/l increase in maternal glucose level at OGTT: fasting glucose 0.31 mmol/l, 1 h glucose 1.58 mmol/l and 2 h glucose 1.27 mmol/l. The 15th percentile cut-offs were determined from the study population distribution

Model 1: offspring’s age and offspring’s sex (male/female)

Model 2: model 1 + maternal age, maternal pre-pregnancy BMI, maternal education level (higher/lower), parity (primiparity/multipara) and maternal smoking (yes/no)

Model 3: model 2 + offspring’s BMI, offspring’s physical activity (MVPA ≥150 min/week or not) and total DQI-I

^*^p<0.05 (after adjusted using the Benjamini–Hochberg procedure with FDR = 0.05)

## Discussion

In this 18-year birth cohort study, our main findings include a consistent association between maternal plasma glucose in pregnancy, analysed as a continuous variable, and offspring’s metabolic outcomes and adiposity traits in early adulthood. Although offspring of mothers with GDM showed trends towards higher metabolic risks, not all associations reached statistical significance. We also observed a significant association between maternal plasma glucose levels during pregnancy and offspring’s indices of insulin secretion evaluated in early adulthood.

### Long-term associations of maternal hyperglycaemia: comparisons with other studies

The long-term association between maternal glucose levels and offspring’s health outcomes has been well documented across different populations and time periods [15]. Evidence linking maternal hyperglycaemia during pregnancy to children’s metabolic health was first documented in the Pima Native American population, where maternal hyperglycaemia during pregnancy was related to fetal programming in a population with notably high rates of obesity and type 2 diabetes [16, 17]. These early findings have since been confirmed by studies in diverse populations, including in Hong Kong cohorts [18].

Regarding glycaemic parameters, our study revealed that maternal plasma glucose in pregnancy, particularly 1 h glucose levels, significantly influenced offspring’s glucose metabolism in early adulthood. While the associations with 1 h glucose levels were particularly strong, the relationships with fasting glucose were relatively weak. This might be partially explained by the larger variability and lower intra-individual consistency in fasting glucose values than in other glycaemic traits, as demonstrated by recent continuous glucose monitoring studies in populations without diabetes [19]. Such inherent variability in fasting glucose measurements could potentially underestimate the true associations between maternal fasting glucose levels and offspring’s outcomes. This finding extends the findings of previous studies showing that offspring exposed to maternal hyperglycaemia face IGT and insulin resistance that persists into adolescence [20]. Our findings provide important clinical insights, highlighting that the impact on offspring’s health occurs across the entire glycaemic spectrum, rather than only above the current diagnostic threshold [21]. Notably, these patterns persisted after adjusting for maternal and offspring’s BMI, suggesting an independent association between maternal glucose levels and offspring’s metabolic health. As demonstrated by McIntyre et al, conventional diagnostic criteria show poor performance in specifically identifying individuals at risk, with receiver operating characteristic (ROC) analyses revealing both the inadequacy of existing classification strategies for hyperglycaemia in pregnancy and the inherent challenge of applying dichotomous approaches to what is essentially a continuous spectrum of risk [22]. These results highlight the potential need for metabolic screening and intervention strategies and for risk stratification that better captures the continuous nature of glycaemic risk rather than focusing solely on GDM diagnosis.

Consistent with previous observational studies that demonstrated an increased risk of childhood obesity in offspring exposed to maternal hyperglycaemia [23, 24], our analysis showed that maternal glucose levels in pregnancy, particularly 1 h and 2 h glucose levels, were positively associated with offspring’s BMI at 18 years. While earlier studies reported that GDM exposure was associated with increased adiposity measures [25], our study found that these associations with long-term adiposity were more evident when analysing maternal plasma glucose as a continuous variable than when using GDM status alone.

Previous animal and epidemiological studies have shown the association between maternal diabetes and offspring’s insulin resistance [26, 27]. A Danish cohort found that intrauterine hyperglycaemia exposure was associated with impairments of glucose metabolism, increased adiposity and an adverse cardiometabolic profile in offspring aged 9–16 years [25]. The persistence of these metabolic alterations into early adulthood is particularly noteworthy, suggesting that intrauterine exposure to hyperglycaemia may programme long-term changes in insulin sensitivity. While adjusting for offspring’s BMI attenuated some of these associations, they remained significant, indicating that the influence is partly independent of adiposity. This observation suggests that maternal hyperglycaemia may have a direct impact on offspring’s metabolic pathways, potentially through epigenetic modifications or altered development of pancreatic beta cells and insulin-sensitive tissues [28]. Moreover, the consistent association with 1 h glucose levels suggests that this time point might be particularly crucial for metabolic programming, offering potential insights for targeted intervention strategies [29].

### Comparisons with earlier observations from the HAPO-FUS

Our 18-year follow-up study extends and differs from the HAPO-FUS conducted at ages 11–14 years in several aspects [7]. While the HAPO-FUS showed associations across all maternal glucose time points, our study identified particularly strong correlations with 1 h glucose levels, suggesting that this time point might be crucial for long-term metabolic programming. The comparison between these two studies at different developmental stages provides valuable insights: although some associations appear to weaken from early adolescence to young adulthood, their persistence at young adulthood demonstrates the lasting impact of maternal plasma glucose on offspring’s metabolic health. This pattern was more pronounced when maternal glucose was examined as a continuous variable rather than using GDM diagnosis alone, indicating the importance of considering the full spectrum of maternal glucose levels in pregnancy.

### Developmental programming of maternal glycaemic influence

Our findings align with and provide further support for the Developmental Origins of Health and Disease (DOHaD) hypothesis, first proposed by David Barker [30] and later expanded by Hanson [31] and others. This concept suggests that the intrauterine environment can substantially influence long-term growth and development outcomes, with both inadequate and excessive nutrition before and during pregnancy contributing to increased risks of obesity, insulin resistance and type 2 diabetes in offspring [32]. The impact of maternal hyperglycaemia on offspring’s metabolic health involves both genetic and environmental factors [33]. Beyond maternal plasma glucose, other maternal factors including obesity and gestational weight gain have been shown to have similar graded effects on offspring’s cardiometabolic outcomes [34]. Our recent genome-wide association study identified several genetic variants associated with gestational diabetes, indicating that genetic predisposition plays an important role in maternal glucose metabolism during pregnancy [35]. The complex interplay between genetic variants and maternal metabolic factors contributes to the developmental programming of offspring’s metabolic health. Understanding these multiple pathways could help identify high-risk mothers and develop targeted intervention strategies.

The persistence of metabolic alterations into early adulthood in our cohort aligns with mechanistic insights from experimental studies. Early rodent experiments revealed the multigenerational impact of maternal hyperglycaemia. In these studies, maternal diabetes and experimentally induced hyperglycaemia were found to result in asymmetrical growth patterns, increased insulin secretion and hyperplasia of insulin-producing beta cells during fetal development, followed by impaired insulin secretion in later life [36]. Mice models of GDM have further shown that even transient glucose intolerance during pregnancy can programme increased adiposity and altered insulin sensitivity in offspring’s adipose tissue [37]. A better understanding of the pathophysiology and heterogeneity of GDM will help to develop targeted interventions with a focus on improved prevention of maternal and offspring’s complications across different life stages, from pre-conception, throughout pregnancy, and beyond [38].

### Strengths and limitations

The strengths of our study include that it was conducted as part of the HAPO study, a blinded observational study in which both clinicians and participants were unaware of maternal glucose levels, thus eliminating potential confounding from treatment interventions. The extended follow-up to about 18 years allowed long-term metabolic consequences to be assessed, addressing a crucial gap in understanding the persistence of these associations into early adulthood. Our comprehensive metabolic evaluations, including detailed measures of glucose metabolism and adiposity, provided a thorough assessment of metabolic health. Additionally, our analysis of maternal glucose as a continuous variable provided important clinical implications, suggesting that even a modest increase in the glycaemic level that is below the IADPSG diagnostic criteria for GDM in maternal glucose levels may influence offspring’s metabolic health, regardless of GDM diagnosis.

This study has several limitations. First, approximately two-thirds of participants were lost to follow-up over 18 years, which might introduce selection bias. A comparison of key characteristics between those who completed follow-up and those who were lost to follow-up showed no significant difference for most variables, suggesting minimal bias in the final model; therefore, the generalisability of our findings may not be affected. Notably, mothers with GDM were slightly more likely to be lost to follow-up than mothers with NGT. While we noticed no significant differences in baseline characteristics between mothers with GDM who were followed up vs those who were lost to follow-up, unmeasured factors might still differ between these groups. Second, our sample size may have been insufficient to detect some clinically meaningful differences, especially in the analyses of categorical outcomes. The limited number of cases of GDM reduced statistical power when using GDM as a categorical exposure, which is why we emphasised continuous glucose measures in our primary analyses. Third, as our study included only Asian women from Hong Kong, the findings may not be applicable to other ethnic populations. Fourth, while we adjusted for many potential confounders, we could not account for all factors that might influence offspring’s outcomes, such as genetic background, familial environment, maternal nutrition and offspring’s psychological stress levels. Fifth, we used OR rather than RR in our analyses, which may overestimate the true associations for outcomes with higher prevalence. Finally, the more robust associations seen with continuous glucose measures than with GDM status may be explained by several methodological limitations. A single OGTT may not adequately reflect the dynamic nature of maternal glycaemic management throughout gestation. In addition, the use of IADPSG criteria for GDM diagnosis, which were primarily based on pregnancy outcomes rather than long-term offspring effects, might not be optimal for identifying mothers whose offspring are at risk of metabolic complications in early adulthood. Further research is needed to determine whether better management of maternal glucose levels during pregnancy could improve long-term offspring health outcomes.

In summary, this 18-year follow-up study demonstrated significant associations between maternal plasma glucose in pregnancy, particularly 1 h glucose levels, and offspring’s metabolic health extending into early adulthood. These associations were most apparent when analysed as continuous traits, suggesting that a metabolic relationship exists across the full range of maternal glucose levels, rather than only above the current diagnostic threshold for GDM. Our findings not only support the Pedersen and DOHaD hypotheses, but also highlight the importance of monitoring and managing maternal glucose levels across the glycaemic spectrum beyond traditional thresholds for GDM screening. The persistence of these associations into early adulthood, despite potential attenuation over time, emphasises the need for glycaemic management during pregnancy. Future studies with more frequent glucose measurements throughout pregnancy may better understand the critical windows for metabolic programming, leading to the development of prevention strategies for complications in offspring’s long-term metabolic health.

## Supplementary Information

Below is the link to the electronic supplementary material.

ESM (PDF 439 KB)

## Data Availability

Data collected for the study will not be made available to others.

## Abbreviations

AGT: Abnormal glucose tolerance
DOHaD: Developmental Origins of Health and Disease
DQI-I: Diet Quality Index-International
FDR: False discovery rate
FPG: Fasting plasma glucose
GDM: Gestational diabetes mellitus
HAPO: Hyperglycemia and Adverse Pregnancy Outcome
HAPO-FUS: Hyperglycemia and Adverse Pregnancy Outcome Follow-up Study
IADPSG: International Association of the Diabetes and Pregnancy Study Groups
IFG: Impaired fasting glucose
IGT: Impaired glucose tolerance
MVPA: Moderate to vigorous intensity physical activity
NGT: Normal glucose tolerance
PG: Plasma glucose
