# Supplementary material for: Association between maternal glucose levels in pregnancy and offspring’s metabolism and adiposity: an 18-year birth cohort study
Source: Diabetologia. 2025 Jul 2;68(10):2205–16. doi: 10.1007/s00125-025-06476-6 (PMC12423139; doi:10.1007/s00125-025-06476-6)
Supplement: Supplementary file 1 — ESM (PDF 439 KB) [file 125_2025_6476_MOESM1_ESM.pdf]

Contents

ESM Table 1. Comparison of baseline characteristics between participants of HAPO follow-up and lost to follow-up at 18 years 2
ESM Table 2. Baseline characteristics of all offspring and mothers at follow-up........ 3
ESM Table 3. Association between maternal GDM and subsequent cardiometabolic outcomes at 18 years postpartum 6
ESM Table 4. Association between maternal glucose level during OGTT and offspring's glucose traits at 18 years old........ 7
ESM Table 5. Association between maternal GDM and offspring's glucose traits at 18 years old 9
ESM Table 6. Association between continuous maternal glucose level and offspring's adiposity traits at 18 years old........ 10
ESM Table 7. Association between maternal GDM and offspring's adiposity traits at 18 years old........ 12
ESM Table 8. Association between continuous maternal glucose level and offspring's insulin sensitivity/β-cell function traits at 18 years old 14
ESM Fig 1. Flow chart describing enrollment for mother–child pairs participating in the whole HAPO study........ 16
ESM Fig 2. Offspring's beta-cell function outcomes at 18 years old across 5 categories of maternal glycaemic levels........ 17

ESM Table 1. Comparison of baseline characteristics between participants of HAPO follow-up and lost to follow-up at 18 years

	HAPO Follow-up at 18 years (n=506)	HAPO lost to follow-up at 18 years (n=1106)	*p* value	SMD
**Maternal characteristics**				
Age, year	31.46±4.45	30.63±5.01	0.001	0.176
Pre-pregnancy BMI (kg/m^2)	21.05±2.88	20.48±2.80	0.0002	0.202
Higher education level	482 (97.77)	1082 (96.21)	0.146	0.087
Systolic blood pressure (SBP, mmHg)	100.95±9.60	101.03±9.78	0.875	0.008
Diastolic blood pressure (DBP, mmHg)	63.22±7.19	63.32±7.29	0.806	0.013
Nulliparity	299 (59.09)	684 (61.79)	0.318	0.056
Family history of diabetes	172 (33.99)	368 (33.24)	0.793	0.017
Smoker	4 (0.79)	30 (2.71)	0.021	0.147

SMD, standardized mean difference.

Continuous variables are expressed as mean ± SD and categorical variables as n (%). Comparisons between groups were performed using Student's t-test for continuous variables and chi-square tests for categorical variables. $P<0.05$ was considered statistically significant.

ESM Table 2. Baseline characteristics of all offspring and mothers at follow-up

	All women (n=506)	Maternal NGT (n=447)	Maternal GDM (n=59)	*p* value
**Maternal baseline characteristics**				
Age (years)	31.46±4.45	31.18±4.44	33.59±3.92	<0.001
Pre-pregnancy BMI (kg/m^2)	21.05±2.88	20.92±2.85	22.08±2.91	0.006
Higher Education level	482 (97.77)	429 (98.62)	53 (91.38)	0.002
Systolic blood pressure (SBP, mmHg)	100.95±9.6	100.73±9.67	102.57±8.96	0.147
Diastolic blood pressure (DBP, mmHg)	63.22±7.19	63.05±7.2	64.48±7.07	0.149
Nulliparity	299 (59.09)	273 (61.07)	26 (44.07)	0.018
Family history of diabetes	172 (33.99)	154 (34.45)	18 (30.51)	0.649
Smoker	4 (0.79)	3 (0.67)	1 (1.69)	0.392
**Maternal characteristic at HAPO 18-year Follow-up Study**				
Age (years)	49.57±4.5	49.3±4.51	51.65±3.89	<0.001
BMI (kg/m^2)	24.06±3.7	24±3.81	24.47±2.73	0.240
Fasting plasma glucose (mmol/l) *	4.9 (0.6)	4.9 (0.6)	5.3 (0.9)	<0.001
HbA_{1c} (mmol/mol) *	37.7 (5.5)	37.7 (5.5)	39.9 (7.7)	<0.001
HbA_{1c} (%) *	5.6 (0.5)	5.6 (0.5)	5.8 (0.7)	<0.001
Fasting plasma insulin (pmol/l) *	57 (58.5)	56.52 (53.7)	76.02 (71.92)	0.020
Waist circumference (cm) *	79.8 (12.15)	79.2 (12.8)	81.3 (10.95)	0.025
Hip circumference (cm) *	97.1 (8.9)	97.25 (9)	96 (7)	0.185
Waist/hip ratio *	0.82 (0.08)	0.81 (0.08)	0.85 (0.09)	<0.001
Body fat percentage (%) *	30.9 (8.05)	30.7 (8.23)	31.9 (6.45)	0.123
Systolic blood pressure (SBP, mmHg)	120.41±15.32	119.84±15.3	124.71±14.88	0.021
Diastolic blood pressure (DBP, mmHg)	78.31±10.61	78.04±10.53	80.34±11.09	0.136
Total cholesterol (TC, mmol/l) *	5.1 (1.3)	5.1 (1.3)	5.2 (1.1)	0.225
Triglyceride (TG, mmol/l) *	0.9 (0.7)	0.9 (0.6)	1.1 (0.9)	0.063

Low-density lipoprotein (LDL, mmol/l) *	3 (1)	3 (1)	3.3 (1.2)	0.093
High-density lipoprotein (HDL, mmol/l) *	1.6 (0.6)	1.6 (0.6)	1.5 (0.4)	0.151
Maternal type 2 diabetes	25 (4.94)	15 (3.36)	10 (16.95)	<0.001
Maternal hypertension	120 (23.72)	100 (22.37)	20 (33.90)	0.073
Maternal hyperlipidaemia	220 (43.48)	189 (42.28)	31 (52.54)	0.176
**Offspring Characteristics at HAPO 18-year Follow-up Study**				
Age (years)	18.09±0.77	18.09±0.77	18.05±0.76	0.674
Sex, male	242 (47.83)	223 (49.89)	19 (32.2)	0.016
Weight (kg)	59.97±13.43	60.08±13.37	59.16±14.01	0.636
Height (cm)	166.4±8.49	166.74±8.43	163.82±8.57	0.016
BMI (kg/m^2)	21.58±4.14	21.53±4.13	21.92±4.21	0.504
Fasting plasma glucose (mmol/l) *	4.6 (0.4)	4.6 (0.4)	4.6 (0.45)	0.874
30-min plasma glucose (mmol/l) *	7.75 (1.7)	7.7 (1.6)	8 (1.65)	0.023
2-hour plasma glucose (mmol/l) *	5.2 (1.6)	5.3 (1.6)	5 (1.9)	0.774
HbA_{1c} (mmol/mol) *	34.4 (3.3)	34.4 (3.3)	34.4 (2.2)	0.440
HbA_{1c} (%) *	5.3 (0.3)	5.3 (0.3)	5.3 (0.2)	0.440
Fasting insulin (pmol/l) *	67.08 (49.62)	67.0 (47.91)	63.3 (46.83)	0.336
30-min insulin (pmol/l) *	719.1 (585.23)	722.4 (594.9)	661.8 (502.44)	0.333
120-min insulin (pmol/l) *	533.22 (542.61)	535.65 (553.18)	513 (342.99)	0.588
Sum of glucose z score *	-0.17(2.85)	-0.15(2.79)	-0.23(3.76)	0.418
Waist Circumference (cm) *	71 (13)	71 (13)	71 (10.95)	0.719
Hip Circumference (cm) *	93.15 (10)	93.1 (9.4)	93.5 (11.65)	0.762
Waist/hip Ratio *	0.77 (0.09)	0.77 (0.09)	0.76 (0.09)	0.647
Body fat percentage (%) *	23.65 (9.92)	23.3 (10.5)	25.5 (6.5)	0.064
HOMA-IR *	2.27 (1.79)	2.27 (1.8)	2.2 (1.63)	0.340
HOMA-B *	214.46 (148.87)	214.56 (146.81)	208.4 (182.49)	0.429
HOMA2-IR *	1.24 (0.86)	1.24 (0.87)	1.24 (0.86)	0.392

HOMA2-B *	134.3 (61.3)	134.85 (60.2)	131.9 (63.3)	0.495
Insulinogenic index *	33.46 (34.01)	34.83 (36.63)	27.54 (24.07)	0.026
Matsuda index *	57.49 (40.68)	57.34 (40.03)	61.65 (41.16)	0.359
Disposition index *	7.5 (0.9)	7.51 (0.91)	7.39 (0.87)	0.124
Systolic blood pressure (SBP, mmHg)	114.3±10.18	114.44±10.01	113.25±11.42	0.448
Diastolic blood pressure (DBP, mmHg)	72.33±7.53	72.39±7.52	71.95±7.62	0.680
Total cholesterol (TC, mmol/l) *	4.1 (0.9)	4.1 (0.97)	4.2 (0.8)	0.466
Triglyceride (TG, mmol/l) *	0.7 (0.4)	0.7 (0.4)	0.8 (0.4)	0.848
Low-density lipoprotein (LDL, mmol/l) *	2.2 (0.8)	2.2 (0.8)	2.2 (0.85)	0.611
High-density lipoprotein (HDL, mmol/l) *	1.5 (0.4)	1.5 (0.4)	1.4 (0.45)	0.531

Continuous variables are expressed as mean ± SD for normally distributed data or median (interquartile range) for non-normally distributed data (marked with *), and categorical variables as n (%). Normally distributed variables were compared using Student's t-test, non-normally distributed variables using Wilcoxon rank-sum test, and categorical variables using chi-square tests. P<0.05 was considered statistically significant.

ESM Table 3. Association between maternal GDM and subsequent cardiometabolic outcomes at 18 years postpartum

		n (%) N=506	Model 1		Model 2	
			OR (95%CI)	*p* value	OR (95%CI)	*p* value
Maternal type 2 diabetes						
	NGT	15 (3.36)	1		1	
	GDM	10 (16.95)	5.17 (2.10, 12.32)	<0.001	5.39 (1.97, 14.17)	0.001
Maternal hypertension						
	NGT	100 (22.37)	1		1	
	GDM	20 (33.90)	1.49 (0.81, 2.68)	0.191	1.26 (0.65, 2.37)	0.477
Maternal hyperlipidaemia						
	NGT	189 (42.28)	1		1	
	GDM	31 (52.54)	1.25 (0.72, 2.19)	0.431	1.20 (0.67, 2.17)	0.536

Odds ratios represent the risk of maternal cardiometabolic outcomes at 18 years postpartum comparing GDM with NGT mothers. Cardiometabolic outcomes were defined as follows: type 2 diabetes (fasting glucose ≥7.0 mmol/L or 2-h glucose ≥11.1 mmol/L, or previous maternal diagnosis of type 2 diabetes, or use of diabetes medication), hypertension (systolic blood pressure ≥140 mmHg or diastolic blood pressure ≥90 mmHg, or previous maternal diagnosis of hypertension, or use of antihypertensive medication), and hyperlipidaemia (total cholesterol ≥6.2 mmol/L or triglycerides ≥2.3 mmol/L or HDL-cholesterol <1.0 mmol/L or LDL-cholesterol ≥4.1 mmol/L, or previous maternal diagnosis of hyperlipidaemia, or use of lipid-lowering medication).

Model 1: Maternal age

Model 2: Model 1 + maternal pre-pregnancy BMI, maternal education level (higher/lower), parity (primiparity/multipara), maternal smoking (yes/no) at OGTT + maternal current BMI

ESM Table 4. Association between maternal glucose level during OGTT and offspring's glucose traits at 18 years old

	n/N (%)	Model 1		Model 2		Model 3	
		OR (95%CI)	*p* value	OR (95%CI)	*p* value	OR (95%CI)	*p* value
**Maternal fasting glucose level at OGTT**							
AGT	42/498 (8.43)	1.07 (0.77, 1.46)	0.680	1.13 (0.81, 1.56)	0.471	1.05 (0.75, 1.45)	0.779
IFG	3/459 (0.65)	0.60 (0.14, 1.92)	0.435	0.65 (0.15, 2.15)	0.532	0.63 (0.14, 2.24)	0.507
IGT	37/493 (7.51)	1.16 (0.83, 1.60)	0.379	1.24 (0.87, 1.74)	0.229	1.14 (0.80, 1.59)	0.466
Fasting glucose level above 85th percentile	76/506 (15.02)	1.26 (0.99, 1.60)	0.056	1.29 (1.00, 1.66)	0.046	1.25 (0.96, 1.62)	0.097
30-min glucose level above 85th percentile	71/498 (14.26)	1.08 (0.84, 1.38)	0.552	1.05 (0.81, 1.37)	0.692	1.00 (0.76, 1.29)	0.971
2-h glucose level above 85th percentile	67/498 (13.45)	1.06 (0.81, 1.36)	0.678	1.12 (0.86, 1.46)	0.397	1.05 (0.79, 1.37)	0.743
Sum of glucose *z* scores above 85th percentile	75/498 (15.06)	1.29 (1.02, 1.64)	0.036*	1.38 (1.07, 1.78)	0.012*	1.35 (1.03, 1.74)	0.024*
**Maternal 1 h glucose level at OGTT**							
AGT	42/498 (8.43)	1.25 (0.92, 1.71)	0.151	1.48 (1.06, 2.08)	0.023*	1.34 (0.94, 1.89)	0.100
IFG	3/459 (0.65)	2.52 (0.89, 7.45)	0.077	2.97 (1.03, 9.04)	0.048	3.37 (1.12, 11.66)	0.032
IGT	37/493 (7.51)	1.15 (0.82, 1.59)	0.420	1.35 (0.94, 1.94)	0.104	1.19 (0.82, 1.73)	0.356
Fasting glucose level above 85th percentile	76/506 (15.02)	1.14 (0.89, 1.46)	0.289	1.24 (0.95, 1.61)	0.108	1.20 (0.91, 1.58)	0.198
30-min glucose level above 85th percentile	71/498 (14.26)	1.44 (1.12, 1.86)	0.005*	1.45 (1.10, 1.91)	0.008*	1.39 (1.04, 1.85)	0.024*
2-h glucose level above 85th percentile	67/498 (13.45)	1.11 (0.86, 1.44)	0.399	1.25 (0.95, 1.64)	0.107	1.18 (0.88, 1.57)	0.273
Sum of glucose *z* scores above 85th percentile	75/498 (15.06)	1.53 (1.20, 1.97)	0.001*	1.75 (1.34, 2.29)	<0.001*	1.84 (1.39, 2.47)	<0.001*
**Maternal 2 h glucose level at OGTT**							
AGT	42/498 (8.43)	1.23 (0.91, 1.67)	0.176	1.42 (1.00, 2.00)	0.046	1.38 (0.97, 1.97)	0.074
IFG	3/459 (0.65)	1.77 (0.61, 5.15)	0.271	2.41 (0.75, 8.44)	0.137	2.56 (0.77, 10.08)	0.131
IGT	37/493 (7.51)	1.17 (0.84, 1.61)	0.335	1.34 (0.93, 1.93)	0.114	1.29 (0.88, 1.88)	0.185
Fasting glucose level above 85th percentile	76/506 (15.02)	1.05 (0.82, 1.34)	0.698	1.16 (0.89, 1.52)	0.274	1.11 (0.83, 1.47)	0.480
30-min glucose level above 85th percentile	71/498 (14.26)	1.24 (0.97, 1.58)	0.089	1.18 (0.90, 1.55)	0.229	1.20 (0.90, 1.59)	0.214
2-h glucose level above 85th percentile	67/498 (13.45)	1.22 (0.94, 1.56)	0.127	1.39 (1.05, 1.83)	0.020	1.35 (1.00, 1.81)	0.048
Sum of glucose *z* scores above 85th percentile	75/498 (15.06)	1.32 (1.03, 1.68)	0.025	1.44 (1.10, 1.87)	0.009*	1.43 (1.08, 1.90)	0.012*

**Maternal sum of all glucose *z* scores**							
AGT	42/498 (8.43)	1.09 (0.96, 1.24)	0.176	1.17 (1.01, 1.34)	0.032	1.12 (0.97, 1.29)	0.120
IFG	3/459 (0.65)	1.22 (0.77, 1.81)	0.355	1.33 (0.83, 2.01)	0.188	1.39 (0.85, 2.26)	0.164
IGT	37/493 (7.51)	1.08 (0.94, 1.24)	0.266	1.16 (0.99, 1.34)	0.058	1.10 (0.94, 1.28)	0.209
Fasting glucose level above 85th percentile	76/506 (15.02)	1.08 (0.97, 1.19)	0.156	1.12 (1.00, 1.26)	0.041	1.10 (0.98, 1.24)	0.112
30-min glucose level above 85th percentile	71/498 (14.26)	1.12 (1.01, 1.24)	0.032	1.11 (0.99, 1.25)	0.067	1.09 (0.97, 1.23)	0.148
2-h glucose level above 85th percentile	67/498 (13.45)	1.07 (0.96, 1.18)	0.239	1.13 (1.01, 1.27)	0.038	1.09 (0.97, 1.23)	0.155
Sum of glucose *z* scores above 85th percentile	75/498 (15.06)	1.18 (1.07, 1.31)	0.001*	1.25 (1.12, 1.41)	<0.001*	1.26 (1.12, 1.42)	<0.001*

Odds ratios represent the risk of offspring's glucose traits for per standard deviation (SD) increase of maternal glucose measures at OGTT: fasting glucose 0.31 mmol/L; 1-h glucose 1.58 mmol/L; 2-h glucose 1.27 mmol/L. The glucose metabolic outcomes were assessed using standard clinical cut-offs: abnormal glucose tolerance (2-h glucose ≥7.8 mmol/L), impaired fasting glucose (fasting glucose 6.1-6.9 mmol/L), and impaired glucose tolerance (2-h glucose 7.8-11.0 mmol/L). The 85th percentile cut-offs were determined from the study population distribution.

Model 1: offspring's age, offspring's sex (male/female)

Model 2: Model 1 + maternal age, maternal pre-pregnancy BMI, maternal education level (higher/lower), parity (primiparity/multipara), and maternal smoking (yes/no)

Model 3: Model 2 + offspring's BMI, offspring's physical activity (MVPA≥150 min/week or not), and total DQI-I

*p<0.05 (after adjusted using the Benjamini–Hochberg procedure with FDR = 0.05)

ESM Table 5. Association between maternal GDM and offspring's glucose traits at 18 years old

	n/N (%)	Model 1		Model 2		Model 3	
		OR	*p* value	OR	*p* value	OR	*p* value
**Offspring's abnormal glucose tolerance**							
NGT	35/439 (7.97)	1		1		1	
GDM	7/59 (11.86)	1.38 (0.53, 3.13)	0.475	1.78 (0.67, 4.25)	0.215	1.45 (0.50, 3.63)	0.459
**Offspring's impaired fasting glucose**							
NGT	2/406 (0.49)	1		1		1	
GDM	1/53 (1.89)	3.57 (0.16, 39.09)	0.308	6.10 (0.25, 84.87)	0.175	6.66 (0.25, 124.18)	0.180
**Offspring's impaired glucose tolerance**							
NGT	31/435 (7.13)	1		1		1	
GDM	6/58 (10.34)	1.33 (0.48, 3.18)	0.549	1.73 (0.60, 4.34)	0.273	1.35 (0.42, 3.62)	0.575

Odds ratios represent the risk of offspring's glucose metabolic traits comparing GDM with NGT mothers. The glucose metabolic outcomes were assessed using standard clinical cut-offs: abnormal glucose tolerance (2 h glucose ≥7.8 mmol/L), impaired fasting glucose (fasting glucose 6.1-6.9 mmol/L), and impaired glucose tolerance (2 h glucose 7.8-11.0 mmol/L).

Model 1: offspring's age, offspring's sex (male/female)

Model 2: Model 1 + maternal age, maternal pre-pregnancy BMI, maternal education level (higher/lower), parity (primiparity/multipara), and maternal smoking (yes/no)

Model 3: Model 2 + offspring's BMI, offspring's physical activity (MVPA≥150 min/week or not), and total DQI-I

ESM Table 6. Association between continuous maternal glucose level and offspring's adiposity traits at 18 years old

	Model 1		Model 2		Model 3	
	β (95%CI)	*p* value	β (95%CI)	*p* value	β (95%CI)	*p* value
**Maternal fasting glucose level at OGTT**						
BMI (kg/m^2) †	0.29 (-0.07, 0.65)	0.113	0.15 (-0.22, 0.51)	0.430	0.18 (-0.20, 0.55)	0.356
Waist circumference (cm)	0.98 (0.10, 1.86)	0.030	0.68 (-0.22, 1.58)	0.140	0.35 (-0.04, 0.74)	0.076
Hip circumference (cm)	0.53 (-0.17, 1.23)	0.136	0.42 (-0.30, 1.14)	0.251	0.17 (-0.21, 0.55)	0.377
Waist/hip ratio	0.006 (0.000, 0.011)	0.045	0.003 (-0.002, 0.009)	0.248	0.002 (-0.003, 0.007)	0.400
Bodyfat percentage (%)	0.60 (-0.07, 1.28)	0.081	0.39 (-0.31, 1.08)	0.274	0.10 (-0.25, 0.45)	0.570
**Maternal 1 h glucose level at OGTT**						
BMI (kg/m^2) †	0.45 (0.09, 0.81)	0.015*	0.42 (0.06, 0.79)	0.024*	0.41 (0.02, 0.79)	0.040
Waist circumference (cm)	1.10 (0.22, 1.97)	0.015*	1.08 (0.18, 1.98)	0.019*	0.18 (-0.23, 0.57)	0.404
Hip circumference (cm)	0.99 (0.29, 1.68)	0.005*	1.04 (0.32, 1.75)	0.005*	0.39 (0.01, 0.77)	0.052
Waist/hip ratio	0.003 (-0.002, 0.009)	0.237	0.003 (-0.003, 0.008)	0.363	-0.001 (-0.006, 0.003)	0.559
Bodyfat percentage (%)	0.82 (0.15, 1.50)	0.017*	0.81 (0.12, 1.51)	0.022*	0.14 (-0.21, 0.50)	0.430
**Maternal 2 h glucose level at OGTT**						
BMI (kg/m^2) †	0.45 (0.09, 0.81)	0.014	0.47 (0.09, 0.84)	0.015*	0.45 (0.06, 0.85)	0.026*
Waist circumference (cm)	1.02 (0.15, 1.90)	0.023	1.15 (0.22, 2.07)	0.015*	0.02 (-0.40, 0.43)	0.951
Hip circumference (cm)	0.63 (-0.06, 1.33)	0.076	0.79 (0.05, 1.53)	0.036	-0.03 (-0.43, 0.37)	0.892
Waist/hip ratio	0.005 (0.000, 0.011)	0.063	0.005 (-0.001, 0.011)	0.093	0.000 (-0.005, 0.005)	0.976
Bodyfat percentage (%)	0.58 (-0.09, 1.26)	0.091	0.65 (-0.07, 1.36)	0.076	-0.12 (-0.49, 0.24)	0.512
**Maternal sum of all glucose *z* scores**						
BMI (kg/m^2) †	0.21 (0.06, 0.36)	0.006*	0.19 (0.03, 0.35)	0.019*	0.19 (0.02, 0.35)	0.026*
Waist circumference (cm)	0.55 (0.18, 0.92)	0.004*	0.54 (0.15, 0.93)	0.007*	0.10 (-0.07, 0.28)	0.241
Hip circumference (cm)	0.38 (0.09, 0.68)	0.011*	0.42 (0.11, 0.73)	0.009*	0.10 (-0.07, 0.27)	0.243
Waist/hip ratio	0.002 (0.000, 0.004)	0.033*	0.002 (0.000, 0.004)	0.108	0.000 (-0.002, 0.002)	0.912
Bodyfat percentage (%)	0.36 (0.07, 0.64)	0.014*	0.34 (0.04, 0.64)	0.026*	0.02 (-0.13, 0.18)	0.751

β values represent the change in offspring's adiposity traits for per standard deviation (SD) increase of maternal glucose measures at OGTT: fasting glucose 0.31 mmol/L; 1-h glucose 1.58 mmol/L; 2-h glucose 1.27 mmol/L.

Model 1: offspring's age, offspring's sex (male/female)

Model 2: Model 1 + maternal age, maternal pre-pregnancy BMI, maternal education level (higher/lower), parity (primiparity/multipara), and maternal smoking (Yes/No)

Model 3: Model 2 + offspring's BMI and offspring's physical activity (MVPA≥150 min/week or not), and total DQI-I

†Analysis was not adjusted for offspring's BMI

*$p<0.05$ (after adjusted using the Benjamini–Hochberg procedure with FDR = 0.05)

ESM Table 7. Association between maternal GDM and offspring's adiposity traits at 18 years old

		n (%) *N* = 504	Model 1		Model 2		Model 3	
			OR (95%CI)	*p* value	OR (95%CI)	*p* value	OR (95%CI)	*p* value
**Overweight/Obesity***								
	NGT	92 (20.67)	1		1		1	
	GDM	14 (23.73)	1.43 (0.72, 2.70)	0.293	1.32 (0.63, 2.64)	0.436	1.29 (0.59, 2.65)	0.504
**Waist circumference above WHO cut-off values**								
	NGT	59 (13.26)	1		1		1	
	GDM	8 (13.56)	1.07 (0.45, 2.28)	0.865	0.94 (0.37, 2.15)	0.897	0.53 (0.09, 2.51)	0.444
**Waist circumference above 85th percentile**								
	NGT	64 (14.38)	1		1		1	
	GDM	9 (15.25)	1.47 (0.63, 3.16)	0.341	1.74 (0.72, 3.90)	0.197	1.96 (0.28, 15.85)	0.508
**Hip circumference above 85th percentile**								
	NGT	64 (14.38)	1		1		1	
	GDM	14 (20.34)	1.81 (0.86, 3.58)	0.100	1.99 (0.92, 4.09)	0.069	3.88 (0.90, 17.34)	0.069
**Waist/hip ratio above WHO cut-off values**								
	NGT	61 (13.71)	1		1		1	
	GDM	10 (16.95)	1.24 (0.68, 2.23)	0.484	1.04 (0.44, 2.26)	0.931	1.11 (0.41, 2.77)	0.831
**Waist/hip ratio above 85th percentile**								
	NGT	69 (15.51)	1		1		1	
	GDM	7 (11.86)	0.94 (0.37, 2.09)	0.879	0.94 (0.36, 2.16)	0.884	0.73 (0.24, 2.01)	0.561

**Bodyfat percentage above WHO cut-off values**								
	NGT	105 (23.60)	1		1		1	
	GDM	13 (22.03)	1.1 (0.54, 2.11)	0.783	1.02 (0.48, 2.03)	0.966	0.71 (0.22, 2.16)	0.555
**Bodyfat percentage above 85th percentile**								
	NGT	68 (15.28)	1		1		1	
	GDM	8 (13.56)	0.76 (0.32, 1.61)	0.505	0.63 (0.24, 1.44)	0.301	0.32 (0.07, 1.21)	0.118

Model 1: offspring's age, offspring's sex (male/female)

Model 2: Model 1 + maternal age, maternal pre-pregnancy BMI, maternal education level (higher/lower), parity (primiparity/multipara), and maternal smoking (Yes/No)

Model 3: Model 2 + offspring's BMI, offspring's physical activity (MVPA≥150 min/week or not), and total DQI-I

[*] Overweight/Obesity was defined as BMI≥24kg/m^2. Analysis was not adjusted for offspring's BMI

All the *p* value ≥ 0.05 after adjusted using the Benjamini–Hochberg procedure with FDR = 0.05

ESM Table 8. Association between continuous maternal glucose level and offspring's insulin sensitivity/β-cell function traits at 18 years old

	Model 1		Model 2		Model 3	
	β (95%CI)	*p* value	β (95%CI)	*p* value	β (95%CI)	*p* value
**Maternal fasting glucose level at OGTT**						
Insulinogenic index	-4.63 (-11.01, 1.75)	0.156	-5.81 (-12.49, 0.87)	0.089	-6.23 (-13.14, 0.68)	0.078
Matsuda index	-1.11 (-4.54, 2.32)	0.525	-1.35 (-4.95, 2.25)	0.463	-0.22 (-3.60, 3.17)	0.900
Disposition index	-0.08 (-0.14, -0.01)	0.024*	-0.09 (-0.16, -0.01)	0.018*	-0.07 (-0.14, 0.00)	0.041*
**Maternal 1 h glucose level at OGTT**						
Insulinogenic index	-6.46 (-12.75, -0.17)	0.045	-6.47 (-13.09, 0.16)	0.056	-8.24 (-15.28, -1.20)	0.022
Matsuda index	-1.44 (-4.84, 1.97)	0.409	-2.27 (-5.86, 1.32)	0.216	-0.12 (-3.60, 3.36)	0.944
Disposition index	-0.13 (-0.19, -0.06)	<0.001*	-0.14 (-0.21, -0.07)	<0.001*	-0.14 (-0.21, -0.07)	<0.001*
**Maternal 2 h glucose level at OGTT**						
Insulinogenic index	-5.81 (-12.25, 0.63)	0.078	-5.44 (-12.37, 1.49)	0.125	-7.31 (-14.64, 0.02)	0.051
Matsuda index	-1.31 (-4.79, 2.16)	0.458	-2.45 (-6.19, 1.30)	0.201	-0.34 (-3.94, 3.27)	0.855
Disposition index	-0.11 (-0.18, -0.04)	0.002*	-0.12 (-0.19, -0.05)	0.002*	-0.11 (-0.18, -0.04)	0.003*
**Maternal sum of all glucose *z* scores**						
Insulinogenic index	-2.97 (-5.64, -0.31)	0.029*	-3.24 (-6.12, -0.37)	0.028*	-3.98 (-7.01, -0.96)	0.010*
Matsuda index	-0.68 (-2.12, 0.76)	0.355	-1.11 (-2.67, 0.45)	0.165	-0.12 (-1.62, 1.37)	0.872
Disposition index	-0.06 (-0.08, -0.03)	<0.001*	-0.06 (-0.09, -0.03)	<0.001*	-0.06 (-0.09, -0.03)	<0.001*

β values represent the change in offspring's β-cell dysfunction/insulin sensitivity measurements for per standard deviation (SD) increase of maternal glucose measures at OGTT: fasting glucose 0.31 mmol/L; 1-h glucose 1.58 mmol/L; 2-h glucose 1.27 mmol/L

Model 1: offspring's age, offspring's sex (male/female)

Model 2: Model 1 + maternal age, maternal pre-pregnancy BMI, maternal education level (higher/lower), parity (primiparity/multipara), and maternal smoking (Yes/No);

Model 3: Model 2 + offspring's BMI, offspring's physical activity (MVPA≥150 min/week or not), and total DQI-I.

*p<0.05 (after adjusted using the Benjamini–Hochberg procedure with FDR = 0.05)

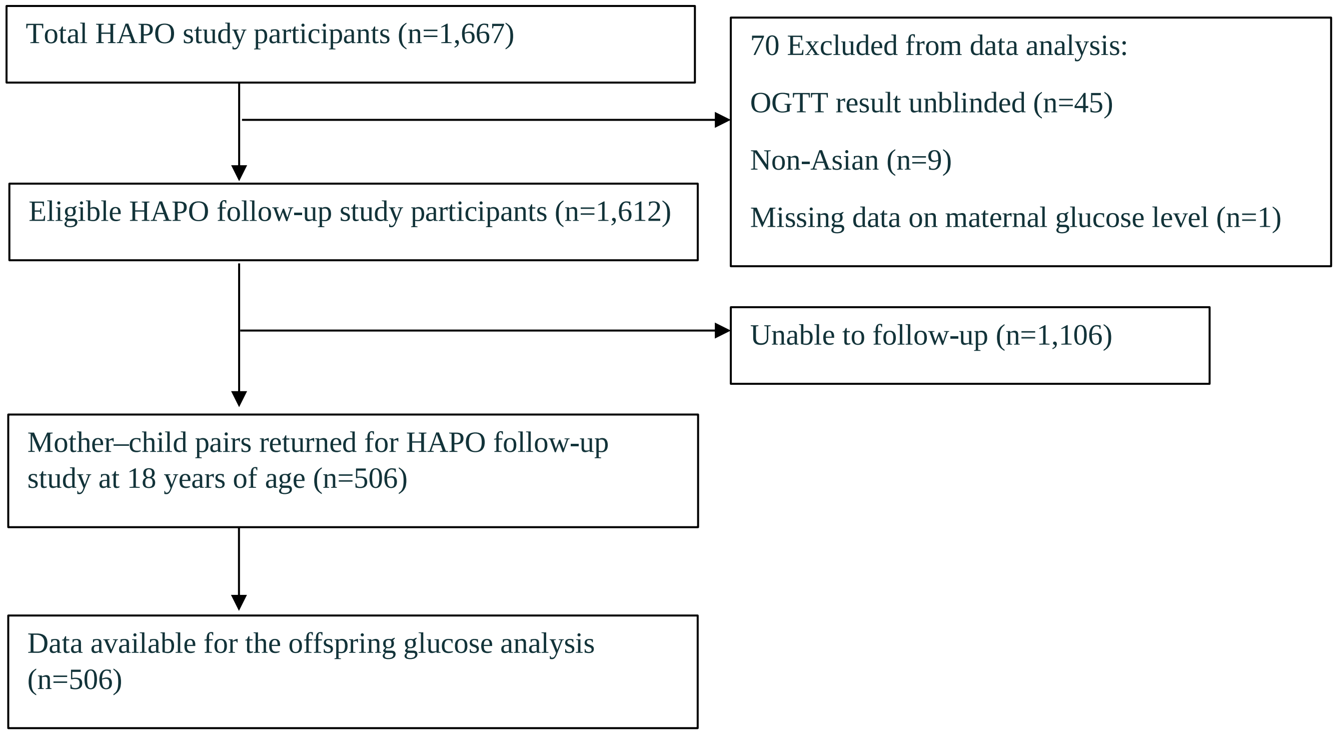

ESM Fig 1. Flow chart describing enrollment for mother–child pairs participating in the whole HAPO study

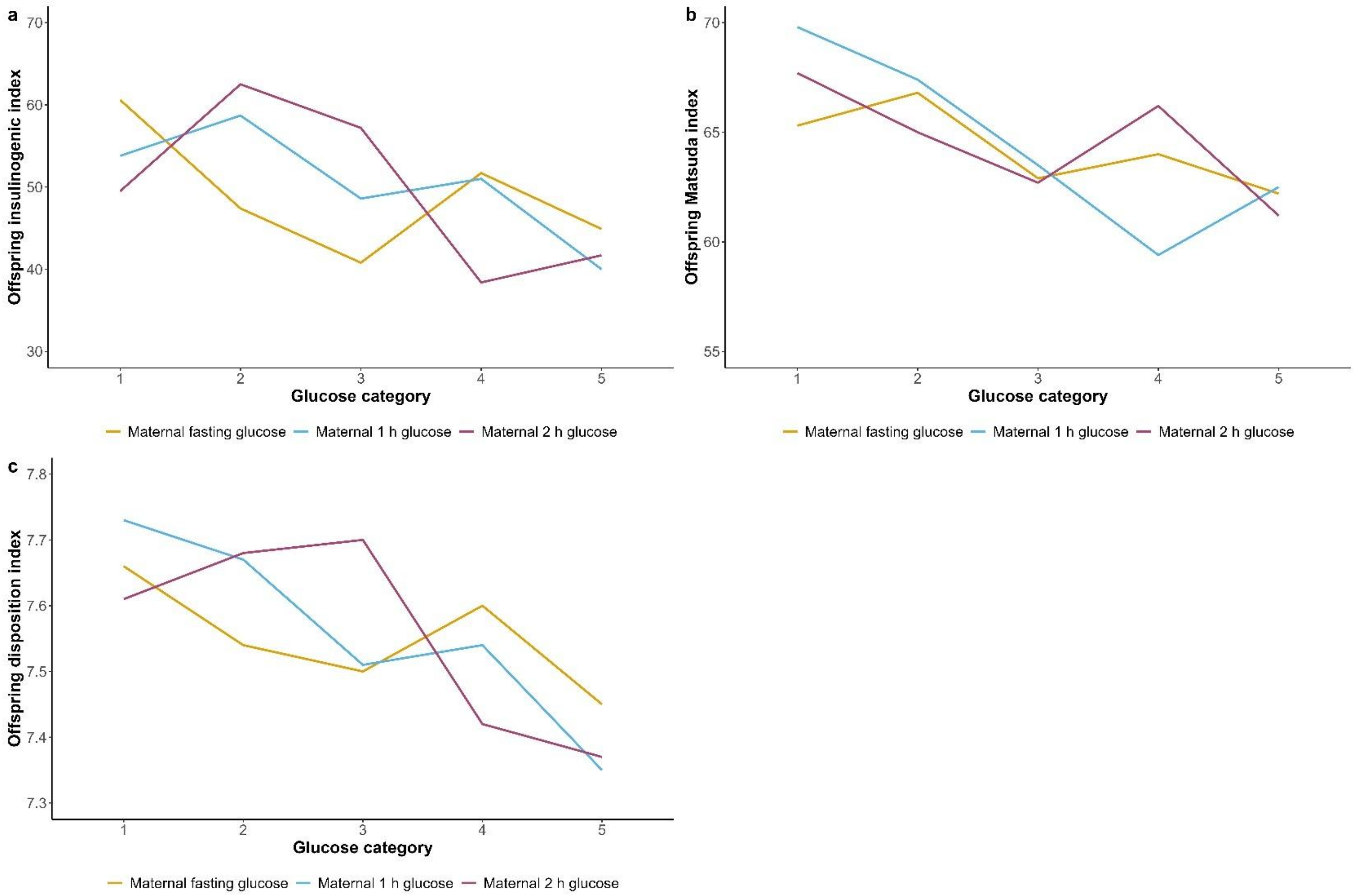

ESM Fig 2. Offspring's beta cell function outcomes at 18 years old across 5 categories of maternal glycaemic levels

Mean levels of offspring's insulinogenic index (a), Matsuda index (b), and disposition index (c) at OGTT across categories of maternal fasting, 1 h and 2 h glucose levels at OGTT in pregnancy
